# Implications of climate change for agricultural productivity in the early twenty-first century

**DOI:** 10.1098/rstb.2010.0158

**Published:** 2010-09-27

**Authors:** Jemma Gornall, Richard Betts, Eleanor Burke, Robin Clark, Joanne Camp, Kate Willett, Andrew Wiltshire

**Affiliations:** Met Office Hadley Centre, FitzRoy Road Exeter, Devon EX1 3PB, UK

**Keywords:** climate, agriculture, food securtity, climate impacts

## Abstract

This paper reviews recent literature concerning a wide range of processes through which climate change could potentially impact global-scale agricultural productivity, and presents projections of changes in relevant meteorological, hydrological and plant physiological quantities from a climate model ensemble to illustrate key areas of uncertainty. Few global-scale assessments have been carried out, and these are limited in their ability to capture the uncertainty in climate projections, and omit potentially important aspects such as extreme events and changes in pests and diseases. There is a lack of clarity on how climate change impacts on drought are best quantified from an agricultural perspective, with different metrics giving very different impressions of future risk. The dependence of some regional agriculture on remote rainfall, snowmelt and glaciers adds to the complexity. Indirect impacts via sea-level rise, storms and diseases have not been quantified. Perhaps most seriously, there is high uncertainty in the extent to which the direct effects of CO_2_ rise on plant physiology will interact with climate change in affecting productivity. At present, the aggregate impacts of climate change on global-scale agricultural productivity cannot be reliably quantified.

## 1. Introduction

Agriculture is strongly influenced by weather and climate. While farmers are often flexible in dealing with weather and year-to-year variability, there is nevertheless a high degree of adaptation to the local climate in the form of established infrastructure, local farming practice and individual experience. Climate change can therefore be expected to impact on agriculture, potentially threatening established aspects of farming systems but also providing opportunities for improvements.

This paper reviews recent literature relevant to the impacts of climate change on global agricultural productivity through a wide range of processes. The aim is to provide a global-scale overview of all relevant impacts, rather than focusing on specific regions or processes, as the purpose of this review is to inform a wider assessment of the risks to global food security. Although there are a large number of studies which focus on the impact of a particular aspect of climate change in a specific location, there are relatively few studies which provide a global assessment. Moreover, these studies tend to focus more on the direct effect of changes in the mean climate state on crop growth and do not consider changes in extremes or in indirect effects of climate change such as sea-level rise or pests and diseases. A comprehensive, internally consistent assessment of all potential direct and indirect effects of climate change on agricultural productivity has not yet been carried out. As a step towards such a full-system assessment, we complement each stage of our review of the literature with presentation of projected changes in relevant climate-related quantities from the Met Office Hadley Centre (MOHC) models. This allows a comparison of the different aspects of climate change relevant to agricultural productivity, so that the relative importance of the different potential causes of impacts can be assessed. This provides some context to decision making in an area of high uncertainty, and also informs future research directions.

Most previous assessments of the impacts of climate change on agriculture (and indeed on other sectors) have focused on time horizons towards the end of the twenty-first century, illustrating the impacts of anthropogenic climate change that could be avoided by reducing greenhouse gas emissions. However, there is also a need to assess the impacts of climate change over the next few decades, which may now be largely unavoidable owing to inertia in the physical climate system and the time scales over which large-scale change in human social, economic and political influences on greenhouse gas emissions could be brought about. Even if greenhouse gas emissions began to be reduced immediately, there would still be some level of ongoing warming for decades and some sea-level rise continuing for centuries, as the climate system is slow to respond fully to imposed changes. There is relatively little information in the literature available on climate change impacts over these time horizons, so we present MOHC climate projections for approximately 2020 and 2050 in order to put the existing literature into context on these time scales.

This paper focuses on impacts on crop productivity, but many of the processes and impacts discussed may also apply to livestock. Some discussion of this is provided in the electronic supplementary material.

## 2. Direct impacts of climate change on agriculture

### (a) Changes in mean climate

The nature of agriculture and farming practices in any particular location are strongly influenced by the long-term mean climate state—the experience and infrastructure of local farming communities are generally appropriate to particular types of farming and to a particular group of crops which are known to be productive under the current climate. Changes in the mean climate away from current states may require adjustments to current practices in order to maintain productivity, and in some cases the optimum type of farming may change.

Higher growing season temperatures can significantly impact agricultural productivity, farm incomes and food security (Battisti & Naylor 2009). In mid and high latitudes, the suitability and productivity of crops are projected to increase and extend northwards, especially for cereals and cool season seed crops (Maracchi *et al*. 2005; Tuck *et al*. 2006; Olesen *et al*. 2007). Crops prevalent in southern Europe such as maize, sunflower and soya beans could also become viable further north and at higher altitudes (Hildén *et al*. 2005; Audsley *et al*. 2006; Olesen *et al*. 2007). Here, yields could increase by as much as 30 per cent by the 2050s, dependent on crop (Alexandrov *et al*. 2002; Ewert *et al*. 2005; Richter & Semenov 2005; Audsley *et al*. 2006; Olesen *et al*. 2007). For the coming century, Fisher *et al*. (2005) simulated large gains in potential agricultural land for the regions such as the Russian Federation, owing to longer planting windows and generally more favourable growing conditions under warming, amounting to a 64 per cent increase over 245 million hectares by the 2080s. However, technological development could outweigh these effects, resulting in combined wheat yield increases of 37–101% by the 2050s (Ewert *et al*. 2005).

Even moderate levels of climate change may not necessarily confer benefits to agriculture without adaptation by producers, as an increase in the mean seasonal temperature can bring forward the harvest time of current varieties of many crops and hence reduce final yield without adaptation to a longer growing season.

**Figure 1.**
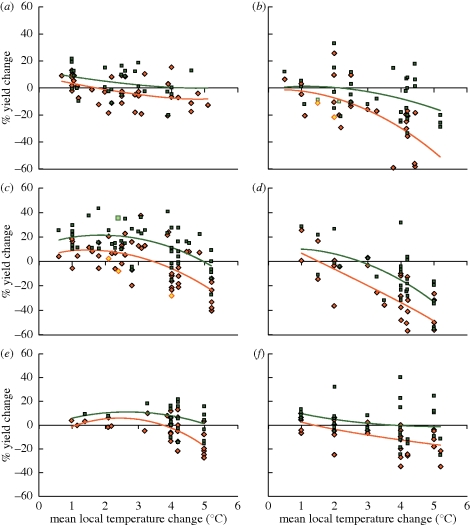
Sensitivity of cereal ((*a*,*b*) maize (mid- to high-latitude and low latitude), (*c*,*d*) wheat (mid- to high-latitude and low latitude) and (*e*,*f*) rice (mid- to high-latitude)) to climate change as determined from the results of 69 studies, against temperature change. Results with (green), and without (red) adaptation are shown. Reproduced from Easterling *et al*. (2007), fig. 5.2.

In areas where temperatures are already close to the physiological maxima for crops, such as seasonally arid and tropical regions, higher temperatures may be more immediately detrimental, increasing the heat stress on crops and water loss by evaporation. A 2°C local warming in the mid-latitudes could increase wheat production by nearly 10 per cent whereas at low latitudes the same amount of warming may decrease yields by nearly the same amount (figure 1). Different crops show different sensitivities to warming. It is important to note the large uncertainties in crop yield changes for a given level of warming (figure 1). By fitting statistical relationships between growing season temperature, precipitation and global average yield for six major crops, Lobell & Field (2007) estimated that warming since 1981 has resulted in annual combined losses of 40 million tonne or US$5 billion (negative relationships between wheat, maize & barley with temperature).

**Figure 2.**
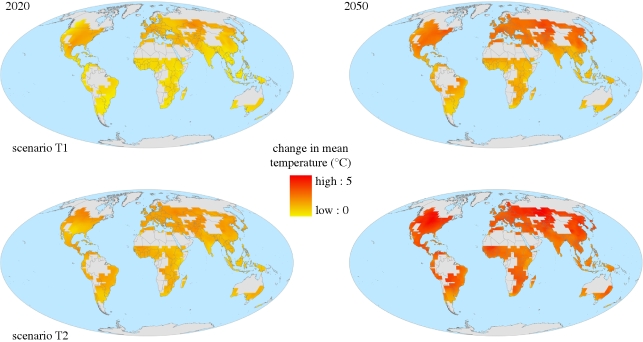
Two projections of change in annual mean temperature (°C) over global croplands for 30-year means centred around 2020 and 2050, relative to 1970–2000. The two projections are the members of the ensemble with the greatest and least change in annual mean temperature averaged over all global croplands. See the electronic supplementary material for further details.

Figure 2 and table 1 show two scenarios for changes in mean annual temperature at 2020 and 2050 relative to present day. All areas of cropland are projected to experience some degree of warming, but the largest change in warming is projected in the high latitudes. However, small increases in temperature in low latitudes may have a greater impact than in high latitudes (figure 1), possibly because agriculture in parts of these regions is already marginal.

**Table 1.** Scenarios of future change in meteorological, hydrological and plant physiological variables relevant to agricultural productivity, selected from an ensemble of 17 scenarios with variants of the HadCM3 climate model. Results are presented as means over global cropland areas for 30-year periods centred on 2020 and 2050, relative to 1970–2000 (except for extreme temperature which is relative to 2000). Two scenarios are presented for each variable, spanning the range of results for each variable to illustrate uncertainties in the projections. For further details see the electronic supplementary material.

	2020	2050
*change in annual mean temperature *(*°C*)**		
scenario T1	1.3	2.8
scenario T2	0.8	1.8
*change in annual mean precipitation *(*mm d^−1^*)**		
scenario P1	0.05	0.05
scenario P2	−0.04	−0.08
*change in 20-year extreme temperature *(*°C*)**		
scenario ET1	1.1	2.9
scenario ET2	0.5	1.7
*change in annual mean net primary productivity *(*kg C m^−2^ y^−1^*)**		
without CO_2_ fertilization	−0.03	−0.07
with CO_2_ fertilization	0.09	0.17
*change in annual mean available crop soil moisture*		
scenario WS1	0.003	0.004
scenario WS2	0.010	0.015
*change in annual mean run-off *(*mm d^−1^*)**		
scenario R1	−0.02	−0.01
scenario R2	0.03	0.07
*change in time spent in drought *(*% of baseline*)**		
scenario D1	11	12
scenario D2	20	22

**Figure 3.**
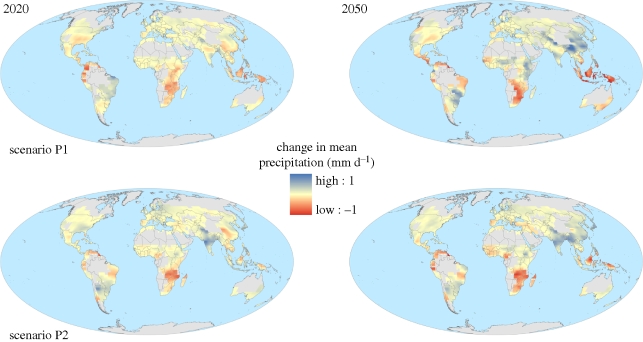
Two projections of change in annual mean precipitation (mm d^−1^) over global croplands for 30-year means centred around 2020 and 2050, relative to 1970–2000. The two projections are the members of the ensemble with the most positive and negative changes in annual mean precipitation averaged over all global croplands. See the electronic supplementary material for further details.

Water is vital to plant growth, so varying precipitation patterns have a significant impact on agriculture. As over 80 per cent of total agriculture is rain-fed, projections of future precipitation changes often influence the magnitude and direction of climate impacts on crop production (Olesen & Bindi 2002; Tubiello *et al*. 2002; Reilly *et al*. 2003). The impact of global warming on regional precipitation is difficult to predict owing to strong dependencies on changes in atmospheric circulation, although there is increasing confidence in projections of a general increase in high-latitude precipitation, especially in winter, and an overall decrease in many parts of the tropics and sub-tropics (IPCC 2007). These uncertainties are reflected in two scenarios shown in figure 3 and table 1, which project different signs of precipitation change averaged over all croplands, even though there is agreement in the sign of change in some regions. One scenario which predicts an overall increase in precipitation, shows large increases in southern USA and India but also significant decreases in the tropics and sub-tropics. The other scenario also shows the decreases in the low latitudes but without significant increases in India.

This reflects the wide range of projections of precipitation change from different climate models (Christensen *et al*. 2007). The differences in precipitation projections arise for a number of reasons. A key factor is the strong dependence on changes in atmospheric circulation which itself depends on the relative rates of warming in different regions, but there are often a number of factors influencing precipitation change projections in a given location. For example, the uncertainty in precipitation change over India arises partly from the expected weakening of the dynamical monsoon circulation (decreasing the Indian monsoon precipitation) versus the increase in atmospheric water content associated with warming (increasing the Indian monsoon precipitation; Meehl *et al*. 2007).

However, changes in seasonal precipitation may be more relevant to agriculture than annual mean changes. In India, climate models generally project a decrease in dry season precipitation and an increase during the rest of the year including the monsoon season, but still with a large inter-model spread (Christensen *et al*. 2007).

Precipitation is not the only influence on water availability. Increasing evaporative demand owing to rising temperatures and longer growing seasons could increase crop irrigation requirements globally by between 5 and 20 per cent, or possibly more, by the 2070s or 2080s (Döll 2002; Fisher *et al*. 2006), but with large regional variations—South-East Asian irrigation requirements could increase by 15 per cent (Döll 2002). Regional studies project increasing irrigation demand in the Middle East and North Africa (Abou-Hadid *et al*. 2003) and potentially 15 per cent increases in irrigation demand in South-East Asia (Arnell *et al*. 2004; Fisher *et al*. 2006). However, decreased requirements are projected in China (Tao *et al*. 2003). Clearly these projections also depend on uncertain changes in precipitation.

### (b) Climate variability and extreme weather events

While change in long-term mean climate will have significance for global food production and may require ongoing adaptation, greater risks to food security may be posed by changes in year-to-year variability and extreme weather events. Historically, many of the largest falls in crop productivity have been attributed to anomalously low precipitation events (Kumar *et al*. 2004; Sivakumar *et al*. 2005). However, even small changes in mean annual rainfall can impact on productivity. Lobell & Burke (2008) report that a change in growing season precipitation by one standard deviation can be associated with as much as a 10 per cent change in production (e.g. millet in South Asia).

For example, Indian agriculture is highly dependent on the spatial and temporal distribution of monsoon rainfall (Kumar *et al*. 2004). Asada & Matsumoto (2009) analysed the relationship between district-level crop yield data (rainy season ‘kharif’ rice) and precipitation for 1960–2000. It was shown that different regions were sensitive to precipitation extremes in different ways. Crop yield in the upper Ganges basin is linked to total precipitation during the relatively short growing season and is thus sensitive to drought. Conversely, the lower Ganges basin was sensitive to pluvial flooding and the Brahmaputra basin demonstrated an increasing effect of precipitation variability on crop yield, in particular drought. These relationships were not consistent through time, in part owing to precipitation trends. Variation between districts implied the importance of social factors and the introduction of irrigation techniques.

Meteorological records suggest that heatwaves became more frequent over the twentieth century, and while individual events cannot be attributed to climate change, the change in probability of a heatwave can be attributed. Europe experienced a particularly extreme climate event during the summer of 2003, with average temperatures 6°C above normal and precipitation deficits of up to 300 mm. A record crop yield loss of 36 per cent occurred in Italy for corn grown in the Po valley where extremely high temperatures prevailed (Ciais *et al*. 2005). It is estimated that such summer temperatures in Europe are now 50 per cent more likely to occur as a result of anthropogenic climate change (Stott *et al*. 2004).

As current farming systems are highly adapted to local climate, growing suitable crops and varieties, the definition of what constitutes extreme weather depends on geographical location. For example, temperatures considered extreme for grain growers in the UK would be considered normal for cereal growers in central France. In many regions, farming may adapt to increases in extreme temperature events by moving to practices already used in warmer climate, for example by growing more tolerant crops. However, in regions where farming exists at the edge of key thresholds increases in extreme temperatures or drought may move the local climate into a state outside historical human experience. In these cases it is difficult to assess the extent to which adaptation will be possible.

#### (i) Extreme temperatures

Recent increases in climate variability may have affected crop yields in countries across Europe since around the mid-1980s (Porter & Semenov 2005) causing higher inter-annual variability in wheat yields. This study suggested that such changes in annual yield variability would make wheat a high-risk crop in Spain. Even mid-latitude crops could suffer at very high temperatures in the absence of adaptation. In 1972, extremely high summer averaged temperature in the former Soviet Union (USSR) contributed to widespread disruptions in world cereal markets and food security (Battisti & Naylor 2009).

Changes in short-term temperature extremes can be critical, especially if they coincide with key stages of development. Only a few days of extreme temperature (greater that 32°C) at the flowering stage of many crops can drastically reduce yield (Wheeler *et al*. 2000). Crop responses to changes in growing conditions can be nonlinear, exhibit threshold responses and are subject to combinations of stress factors that affect their growth, development and eventual yield. Crop physiological processes related to growth such as photosynthesis and respiration show continuous and nonlinear responses to temperature, while rates of crop development often show a linear response to temperature to a certain level. Both growth and developmental processes, however, exhibit temperature optima. In the short-term high temperatures can affect enzyme reactions and gene expression. In the longer term these will impact on carbon assimilation and thus growth rates and eventual yield. The impact of high temperatures on final yield can depend on the stage of crop development. Wollenweber *et al*. (2003) found that the plants experience warming periods as independent events and that critical temperatures of 35°C for a short-period around anthesis had severe yield reducing effects. However, high temperatures during the vegetative stage did not seem to have significant effects on growth and development. Reviews of the literature (Porter & Gawith 1999; Wheeler *et al*. 2000) suggest that temperature thresholds are well defined and highly conserved between species, especially for processes such as anthesis and grain filling.

Although groundnut is grown in semi-arid regions which regularly experience temperatures of 40°C, if after flowering the plants are exposed to temperatures exceeding 42°C, even for short periods, yield can be drastically reduced (Vara Prasad *et al*. 2003). Maize exhibits reduced pollen viability for temperatures above 36°C. Rice grain sterility is brought on by temperatures in the mid-30s and similar temperatures can lead to the reverse of the vernalizing effects of cold temperatures in wheat. Increases in temperature above 29°C for corn, 30°C for soya bean and 32°C for cotton negatively impact on yields in the USA.

**Figure 4.**
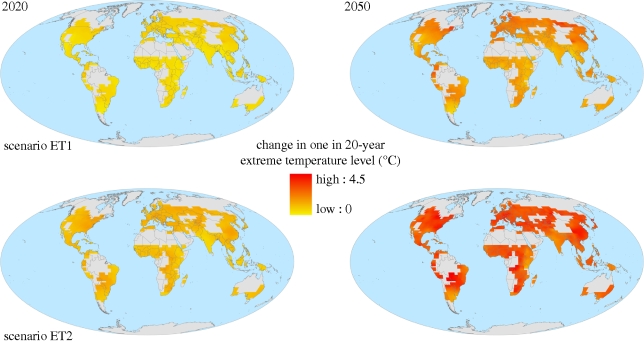
Two projections of change in one in 20-year extreme temperature level (°C) over global croplands for 2020 and 2050, relative to 2000. The two projections are the members of the ensemble with the greatest and least change averaged over all global croplands. See the electronic supplementary material for further details.

Figure 4 and table 1 show that in all cases and all regions, one in 20-year extreme temperature events is projected to be hotter. Events which today are considered extreme would be less unusual in the future. The impacts of extreme temperature events can be difficult to separate from those of drought. However, key temperature thresholds exist beyond which crop physiology is altered, potentially devastating yields.

#### (ii) Drought

There are a number of definitions of drought, which generally reflect different perspectives. Holton *et al*. (2003) point out that ‘the importance of drought lies in its impacts. Thus definitions should be region-specific and impact- or application-specific in order to be used in an operational mode by decision makers.’ It is common to distinguish between meteorological drought (broadly defined by low precipitation), agricultural drought (deficiency in soil moisture, increased plant water stress), hydrological drought (reduced streamflow) and socio-economic drought (balance of supply and demand of water to society; Holton *et al*. 2003). Globally, the areas sown for the major crops of barley, maize, rice, sorghum, soya bean and wheat have all seen an increase in the percentage of area affected by drought as defined in terms of the Palmer Drought Severity Index (PDSI; Palmer 1965) since the 1960s, from approximately 5–10% to approximately 15–25% (Li *et al*. 2009). Global mean PDSI has also increased (IPCC 2007), and a comparison of climate model simulations with observed data suggests that anthropogenic increases in greenhouse gas and aerosol concentrations have made a detectable contribution to the observed drying trend in PDSI (Burke *et al*. 2006).

In climate-modelling studies, Burke *et al*. (2006) define drought as the 20th percentile of the PDSI distribution over time, for pre-industrial conditions; this definition is therefore regionally specific. Therefore at any given time, approximately 20 per cent of the land surface will be defined as being in drought, but the conditions in a normally wet area under drought may still be less dry than those in another region which is dry under normal conditions. Using this definition, the MOHC climate model simulates the proportion of the land surface under drought to have increased from 20 to 28 per cent over the twentieth century (Burke *et al*. 2006).

Li *et al*. (2009) define a yield reduction rate (YRR) which takes a baseline of the long-term trend in yield (assumed to be owing to technological progress and infrastructure improvement) and compares this with actual annual yields to define a YRR owing to climate variability. Using national-scale data for the four major grains (barley, maize, rice and wheat), Li *et al*. (2009) suggested that 60–75% of observed YRRs can be explained by a linear relationship between YRR and a drought risk index based on the PDSI. Present-day mean YRR values are diagnosed as ranging from 5.82 per cent (rice) to 11.98 per cent (maize). By assuming the linear relationship between the drought risk index and YRR holds into the future, Li *et al*. (2009) estimated that drought related yield reductions would increase by more than 50 per cent by 2050 for the major crops.

The impacts of drought may offset benefits of increased temperature and season length observed at mid to high latitudes. Using models of global climate, crop production and water resources, Alcamo *et al*. (2007) suggested that decreased crop production in some Russian regions could be compensated by increased production in others, resulting in relatively small average changes. However, their results indicate that the frequency of food production shortfalls could double in many of the main crop growing areas in the 2020s, and triple in the 2070s (Alcamo *et al*. 2007). Although water availability in Russia is increasing on average, the water resources model predicted more frequent low run-off events in the already dry crop growing regions in the south, and a significantly increased frequency of high run-off events in much of central Russia (Alcamo *et al*. 2007).

#### (iii) Heavy rainfall and flooding

**Figure 5.**
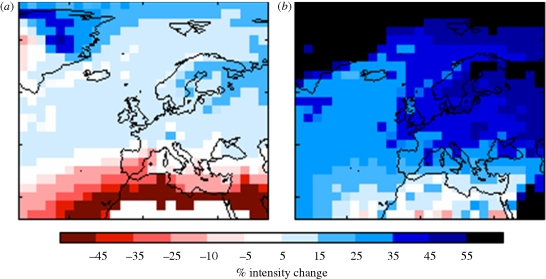
(*a*) Lower and (*b*) upper estimates covering the central 80% range of changes in precipitation intensity on wet days with a 1 year return period for a doubling of CO_2_.

Food production can also be impacted by too much water. Heavy rainfall events leading to flooding can wipe out entire crops over wide areas, and excess water can also lead to other impacts including soil water logging, anaerobicity and reduced plant growth. Indirect impacts include delayed farming operations (Falloon & Betts in press). Agricultural machinery may simply not be adapted to wet soil conditions. In a study looking at the impacts of current climate variability, Kettlewell *et al*. (1999) showed that heavy rainfall in August was linked to lower grain quality which leads to sprouting of the grain in the ear and fungal disease infections of the grain. This was shown to affect the quality of the subsequent products such that it influenced the amount of milling wheat that was exported from the UK. The proportion of total rain falling in heavy rainfall events appears to be increasing, and this trend is expected to continue as the climate continues to warm. A doubling of CO_2_ is projected to lead to an increase in intense rainfall over much of Europe. In the higher end projections, rainfall intensity increases by over 25 per cent in many areas important for agriculture (figure 5).

#### (iv) Tropical storms

A tropical cyclone is the generic term for a non-frontal synoptic scale low-pressure system over tropical or sub-tropical waters with organized convection (i.e. thunderstorm activity) and definite cyclonic surface wind circulation (Holland 1993). Severe tropical cyclones, with maximum sustained wind speeds of at least 74 mph, are known as ‘hurricanes’ in the eastern North Pacific and North Atlantic and ‘typhoons’ in the western North Pacific. The strongest tropical cyclones can reach wind speeds as large as 190 mph, as recorded in Typhoon Tip in the western North Pacific in October 1979. Tropical cyclones usually occur during the summer and early autumn: around May–November in the Northern Hemisphere and November–April in the Southern Hemisphere, although tropical cyclones are observed all year round in the western North Pacific. The North Indian Ocean is the only basin to have a two-part tropical cyclone season: before and after the onset of the South Asian monsoon, from April to May and October to November, respectively.

**Figure 6.**
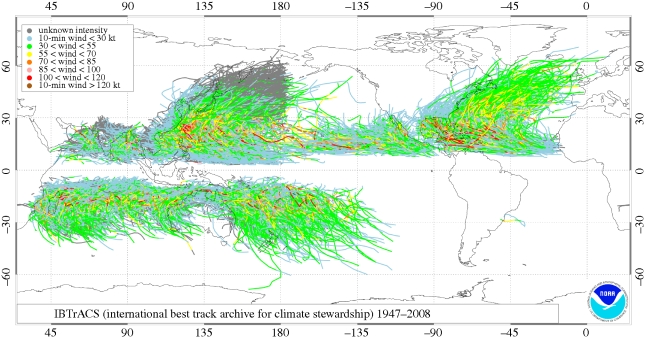
Observed tropical cyclone tracks and intensity for all known storms over the period 1947–2008. Tracks are produced from the IBTrACS dataset of NOAA/NCDC (Knapp *et al*. 2010).

Figure 6 shows observed tropical cyclone tracks for all known storms over the period 1945–2008. In this context, the most vulnerable agricultural regions are found, among others, in the USA, China, Vietnam, India, Bangladesh, Myanmar and Madagascar.

Both societal and economic implications of tropical cyclones can be high, particularly in developing countries with high population growth rates in vulnerable tropical and subtropical regions. This is particularly the case in the North Indian Ocean, where the most vulnerable people live in the river deltas of Myanmar, Bangladesh, India and Pakistan; here population growth has resulted in increased farming in coastal regions most at risk from flooding (Webster 2008). In 2007, cyclone Sidr hit Bangladesh costing 3500 lives (United Nations 2007), and in 2008 cyclone Nargis caused 130 000 deaths in Myanmar. The agricultural impacts of these and other recent cyclones are shown in table 2.

**Table 2.** Selected tropical cyclones of the past decade, and their agricultural impacts.

date	location	cyclone name	agricultural impact
Feb–Apr 2000	Madagascar	Eline, Gloria (Feb), Hudah (Apr)	combined losses owing to three cyclones: 149 441 hectares rice (7% of annual production), 5000 hectares maize, 155 000 hectares cereals (FAO 2000)
2006–2007	Madagascar	Bondo (Dec 2006), Clovis (Jan 2007), Favio (Jan 2007), Gamede (Feb 2007), Indlala (Mar 2007)	combined losses: 90 000 hectares of crop (IFRC 2007); 80% of vanilla production lost to Indlala alone (FAO 2007)
2007	Mozambique	Favio	thousands of hectares of crop destroyed (FAO 2007)
Nov 2007	Bangladesh	Sidr	1.6 million acres of cropland damaged; >25% winter rice crop destroyed (United Nations 2007)
May 2008	Irrawaddy Delta, Myanmar (Burma)	Nargis	estimated 4 m storm surge inundated coastal areas and regions up to 40 km inland (Webster 2008). Soil salination made 50 000 acres of rice cropland now unfit for planting (Stover & Vinck 2008). Loss of rice seed, fertilizers, farm machinery, and valuable land threatened the winter 2008/09 rice crop including exports to neighbouring countries (FAO 2009)

Although many studies focus on the negative impacts, tropical cyclones can also bring benefits. In many arid regions in the tropics, a large portion of the annual rain comes from cyclones. It is estimated that tropical cyclones contribute to 15–20% of South Florida's annual rainfall (Walther & Abtew 2006), which can temporarily end severe regional droughts. Examples of such storms are hurricane Gabrielle (2001) and tropical storm Fay (2008), which provided temporary relief from the 2000–2001 and 2006–2009 droughts, respectively. As much as 15 inches of rainfall was recorded in some regions from tropical storm Fay, without which, regions would have faced extreme water shortage, wildfires and potential saltwater intrusion into coastal freshwater aquifers (Abtew *et al*. 2009). Tropical cyclones can also help replenish water supplies to inland regions: cyclone Eline, which devastated agriculture in Madagascar in February 2000, later made landfall in southern Africa and contributed significantly to the rainfall in the semi-desert region of southern Namibia.

There is much debate on the global change in tropical cyclone frequency and intensity under a warming climate. Climate modelling studies contributing to the IPCC's Fourth Assessment Report (AR4) suggest tropical cyclones may become more intense in the future with stronger winds and heavier precipitation (Meehl *et al*. 2007). This is in agreement with more recent studies using high resolution models, which also indicate a possible decrease in future global tropical cyclone frequency (McDonald *et al*. 2005; Bengtsson *et al*. 2007; Gualdi *et al*. 2008). However, there is limited consensus among the models on the regional variations in tropical cyclone frequency.

## 3. Indirect impacts of climate change on agricultural productivity

### (a) Pests and diseases

Rising atmospheric CO_2_ and climate change may also impact indirectly on crops through effects on pests and disease. These interactions are complex and as yet the full implications in terms of crop yield are uncertain. Indications suggest that pests, such as aphids (Newman 2004) and weevil larvae (Staley & Johnson 2008), respond positively to elevated CO_2_. Increased temperatures also reduced the overwintering mortality of aphids enabling earlier and potentially more widespread dispersion (Zhou *et al*. 1995). Evidence suggests that in sub-Saharan Africa migration patterns of locusts may be influenced by rainfall patterns (Cheke & Tratalos 2007) and thus potential exists for climate change to shape the impacts of this devastating pest. Pathogens and disease may also be affected by a changing climate. This may be through impacts of warming or drought on the resistance of crops to specific diseases and through the increased pathogenicity of organisms by mutation induced by environmental stress (Gregory *et al*. 2009). Over the next 10–20 years, disease affecting oilseed rape could increase in severity within its existing range as well as spread to more northern regions where at present it is not observed (Evans *et al*. 2008). Changes in climate variability may also be significant, affecting the predictability and amplitude of outbreaks.

### (b) Changes in water availability owing to remote climate changes

Climate changes remote from production areas may also be critical. Irrigated agricultural land comprises less than one-fifth of all cropped area but produces between 40 and 45 per cent of the world's food (Döll & Siebert 2002), and water for irrigation is often extracted from rivers which depend upon distant climatic conditions. For example, agriculture along the Nile in Egypt depends on rainfall in the upper reaches of the Nile such as the Ethiopian Highlands.

**Figure 7.**
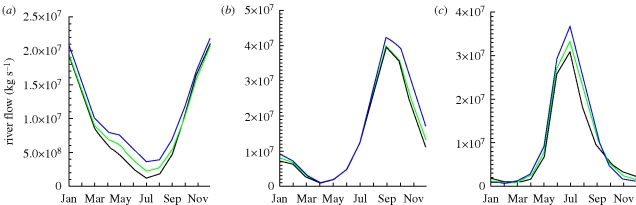
Projected mean monthly river flow (kg s^−1^) for 30 year means centred on 2000 (black), 2020 (green) and 2050 (blue) for the (*a*) Nile, (*b*) Ganges and (*c*) Volga. Projections are bias corrected ensemble means from the Hadley Centre models. See the electronic supplementary material for further details.

Figure 7 shows the projected changes in monthly river-flow for the 2020s and 2050s for selected key rivers of interest in this context. In some rivers such as the Nile, climate change increases flow throughout the year which could confer benefits to agriculture. However, in other catchments, e.g. the Ganges, the increase in run-off comes as an increase in peak flow around the monsoon. However, dry season river-flow is still very low. Without sufficient storage of peak season flow, water scarcity may affect agricultural productivity despite overall increases in annual water availability. Increases at peak flow may also cause damage to crop lands through flooding.

**Figure 8.**
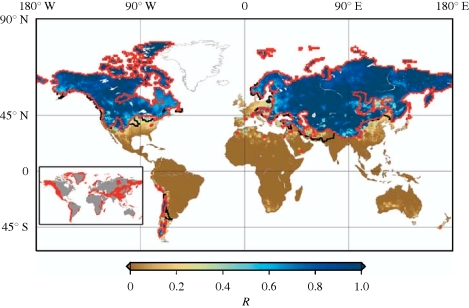
The fraction of run-off originating as snowfall. The red lines indicate the regions where streamflow is snowmelt-dominated, and where there is not adequate reservoir storage capacity to buffer shifts in the seasonal hydrograph. The black lines indicate additional areas where water availability is predominantly influenced by snowmelt generated upstream (but run-off generated within these areas is not snowmelt-dominated). Reproduced from Barnett *et al*. (2005) with permission from Macmillan Publishers Ltd: Nature.

Figure 8 shows areas in the world where river flow is dominated by snow melt. These areas are mostly at mid to high latitudes where predictions for warming are greatest. Warming in winter means that less precipitation falls as snow and that which accumulates melts earlier in the year. Changing patterns of snow cover fundamentally alter how such systems store and release water. Changes in the amount of precipitation affect the volume of run-off, particularly near the end of the winter at the onset of snow melt. Temperature changes mostly affect the timing of run-off with earlier peak flow in the spring. Although additional river-flow can be considered beneficial to agriculture this is only true if there is an ability to store run-off during times of excess to use later in the growing season. Globally, only a few rivers currently have adequate storage to cope with large shifts in seasonality of run-off (Barnett *et al*. 2005). Where storage capacities are not sufficient, much of the winter run-off will immediately be lost to the oceans. Figure 7*c* shows the monthly river-flow from the Volga catchment in Russia. It shows an earlier and increased peak flow around snow melt with subsequently lower flow later in the year.

Some major rivers, such as the Indus and Ganges, are fed by mountain glaciers, with approximately one-sixth of the world's population currently living in glacier-fed river basins (Stern 2007). Populations are projected to rise significantly in major glacier-fed river basins such as the Indo-Gangetic plain. As such, changes in remote precipitation and the magnitude and seasonality of glacial melt waters could therefore potentially impact food production for many people.

The majority of observed glaciers around the globe are undergoing shrinkage (Zemp *et al.* 2008). Formerly attributing this retreat to recent warming is not currently possible. However, there is a broad consensus that warming is a primary cause of retreat, although changes in atmospheric moisture particularly in the tropics may be contributing (Bates *et al*. 2008). Melting glaciers will initially increase river-flow although the seasonality of flow will be enhanced (Juen *et al*. 2007) bringing with it an increased flood risk. In the long term, glacial retreat is expected to be enhanced further leading to eventual decline in run-off, although the greater time scale of this decline is uncertain. The Chinese Glacier Inventory catalogued 46 377 glaciers in western China, with approximately 15 000 glaciers in the Himalayas. In total these glaciers store an estimated 12 000 km^3^ of fresh water (Ding *et al*. 2006; Cruz *et al*. 2007). Analysis of glaciers in the western Himalayas demonstrates evidence of glacial thinning (Berthier *et al*. 2007), and radioactive bomb deposits from one high altitude glacier show no net accumulation since 1950 (Kehrwald *et al*. 2008). The limited number of direct observations also supports evidence of a glacial retreat in the Himalayas (Zemp *et al.* 2008). The water from these glaciers feeds large rivers such as the Indus, Ganges and Brahmaputra and is likely to be contributing a significant proportion of seasonal river flow although the exact magnitude is unknown. Currently nearly 500 million people are reliant on these rivers for domestic and agricultural water resources. Climate change may mean the Indus and Ganges become increasingly seasonal rivers, ceasing to flow during the dry season (Kehrwald *et al*. 2008). Combined with a rising population this means that water scarcity in the region would be expected to increase in the future.

### (c) Mean sea-level rise

Sea-level rise is an inevitable consequence of a warming climate owing to a combination of thermal expansion of the existing mass of ocean water and addition of extra water owing to the melting of land ice. This can be expected to eventually cause inundation of coastal land, especially where the capacity for introduction or modification of sea defences is relatively low or non-existent. Regarding crop productivity, vulnerability is clearly greatest where large sea-level rise occurs in conjunction with low-lying coastal agriculture. Many major river deltas provide important agricultural land owing to the fertility of fluvial soils, and many small island states are also low-lying. Increases in mean sea level threaten to inundate agricultural lands and salinize groundwater in the coming decades to centuries, although the largest impacts may not be seen for many centuries owing to the time required to melt large ice sheets and for warming to penetrate into the deep ocean.

The potential sea-level rise associated with melting of the main ice sheets would be 5 m for West Antarctic Ice Sheet (WAIS), 60 m for East Antarctic Ice Sheet (EAIS), and 7 m for Greenland Ice Sheet (GIS), with both the GIS and WAIS considered vulnerable. Due to the possible rate of discharge of these ice sheets, and past maximal sea-level rise (under similar climatic conditions) a maximum eustatic sea-level rise of approximately 2 m by 2100 is considered physically plausible, but very unlikely (Pfeffer *et al*. 2008; Rohling *et al*. 2008; Lowe *et al*. 2009).

Short-lived storm surges can also cause great devastation, even if land is not permanently lost. There has been relatively little work assessing the impacts of either mean sea-level rise or storm surges on agriculture.

## 4. Non-climate impacts related to greenhouse gas emissions: impacts of changes in atmospheric composition

### (a) CO_2_ fertilization

As well as influencing climate through radiative forcing, increasing atmospheric CO_2_ concentrations can also directly affect plant physiological processes of photosynthesis and transpiration (Field *et al*. 1995). Therefore any assessment of the impacts of CO_2_-induced climate change on crop productivity should account for the modification of the climate impact by the CO_2_ physiological impact. The CO_2_ physiological response varies between species, and in particular, two different pathways of photosynthesis (named C_3_ and C_4_) have evolved and these affect the overall response. The difference lies in whether ribulose-1,5-bisphosphate carboxylase–oxygenase (RuBisCO) within the plant cells is saturated by CO_2_ or not. In C_3_ plants, RuBisCO is not CO_2_-saturated in present day atmospheric conditions, so rising CO_2_ concentrations increase net uptake of carbon and thus growth. The RuBisCO enzyme is highly conserved in plants and as such it is thought that the response of all C_3_ crops including wheat and soya beans will be comparable. Theoretical estimates suggest that increasing atmospheric CO_2_ concentrations to 550 ppm, could increase photosynthesis in such C_3_ crops by nearly 40 per cent (Long *et al*. 2004). The physiology of C_4_ crops, such as maize, millet, sorghum and sugarcane is different. In these plants CO_2_ is concentrated to three to six times atmospheric concentrations and thus RuBisCO is already saturated (von Caemmerer & Furbank 2003). Thus, rising CO_2_ concentrations confer no additional physiological benefits. These crops may, however, become more water-use efficient at elevated CO_2_ concentrations as stomata do not need to stay open as long for the plant to receive the required CO_2._ Thus yields may increase marginally as a result (Long *et al*. 2004).

Many studies suggest that yield rises owing to this CO_2_-fertilization effect and these results are consistent across a range of experimental approaches including controlled environment closed chambers, greenhouse, open and closed field top chambers, and free-air carbon dioxide enrichment (FACE) experiments (Tubiello *et al*. 2007). Experiments under idealized conditions show that a doubling of atmospheric CO_2_ concentration increases photosynthesis by 30–50% in C_3_ plant species and 10–25% in C_4_ species (Ainsworth & Long 2005). Crop yield increase is lower than the photosynthetic response; increases of atmospheric CO_2_ to 550 ppm would on average increase C_3_ crop yields by 10–20% and C_4_ crop yields by 0–10% (Gifford 2004; Long *et al*. 2004; Ainsworth & Long 2005).

Some authors argue that crop response to elevated CO_2_ may be lower than previously thought, with consequences for crop modelling and projections of food supply (Long *et al*. 2004, 2009). Plant physiologists and modellers alike recognize that the effects of elevated CO_2_, as measured in experimental settings and subsequently implemented in models, may overestimate actual field and farm level responses. This is because of many limiting factors such as pests and weeds, nutrients, competition for resources, soil water and air quality which are neither well understood at large scales, nor well implemented in leading models.

Despite the potential positive effects on yield quantities, elevated CO_2_ may, however, be detrimental to yield quality of certain crops. For example, elevated CO_2_ is detrimental to wheat flour quality through reductions in protein content (Sinclair *et al*. 2000).

**Figure 9.**
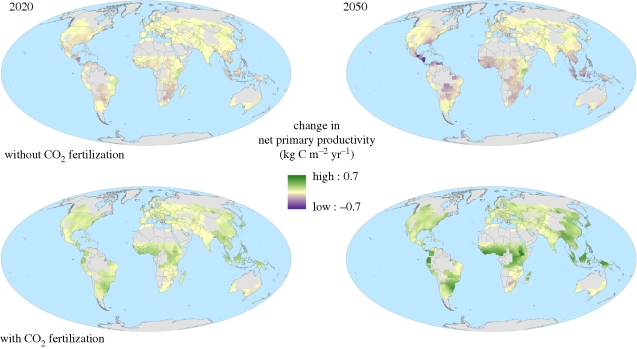
Two projections of future change in net primary productivity (kg C m^−2^ yr^−1^) over global croplands for 30-year means centred around 2020 and 2050, relative to 1970–2000. The two projections show the impact of including CO_2_ physiological effects and are the members of the ensemble with the most positive and negative changes in productivity averaged over all global croplands. See the electronic supplementary material for further details.

Figure 9 and table 1 show the impact of including CO_2_ physiological effects in projections of plant productivity in agricultural regions. Without CO_2_ fertilization, many regions, especially in the low latitudes, suffer a decrease in productivity by 2050. In contrast, by including CO_2_ fertilization all but the very driest regions show increases in productivity.

**Figure 10.**
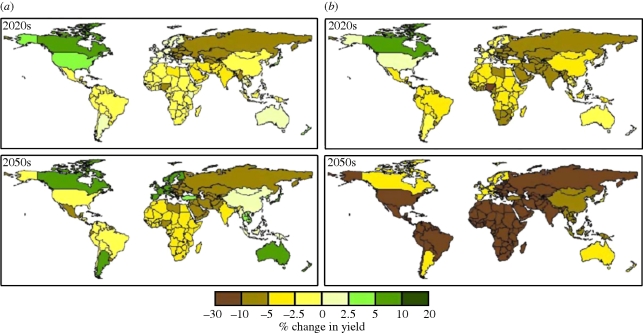
Potential changes (%) in national cereal yields for the 2020s and 2050s relative to 1990, with climate change projected by the HadCM3 model under the A1FI scenario (*a*) with and (*b*) without CO_2_ fertilization. Reproduced from Parry *et al*. (2004) with permission from Elsevier.

Global-scale comparisons of the impacts of CO_2_ fertilization with those of changes in mean climate (Parry *et al*. 2004; Nelson *et al*. 2009) show that the strength of CO_2_ fertilization effects is a critical factor in determining whether global-scale yields are projected to increase or decrease. If CO_2_ fertilization is strong, North America and Europe may benefit from climate change at least in the short term (figure 10). However, regions such as Africa and India are nevertheless still projected to experience up to 5 per cent losses by 2050, even with strong CO_2_ fertilization. These losses increase to up to 30 per cent if the effects of CO_2_ fertilization are omitted. In fact without CO_2_ fertilization all regions are projected to experience a loss in productivity owing to climate change by 2050. However, existing global scale studies (Parry *et al*. 2004; Nelson *et al*. 2009) have only used a limited sample of available climate model projections.

A reduction in CO_2_ emissions would be expected to reduce the positive effect of CO_2_ fertilization on crop yields more rapidly than it would mitigate the negative impacts of climate change. Even if GHG concentrations rose no further, there is a commitment to a certain amount of further global warming (IPCC 2007). Stabilization of CO_2_ concentrations would therefore halt any increase in the impacts of CO_2_ fertilization, while the impacts of climate change could still continue to grow. Therefore in the short term the impacts on global food production could be negative. However, estimates suggest that stabilizing CO_2_ concentrations at 550 ppm would significantly reduce production losses by the end of the century (Arnell *et al*. 2002; Tubiello & Fisher 2006).

For all species higher water-use efficiencies and greater root densities under elevated CO_2_ in field systems may, in some cases, alleviate drought pressures, yet their large-scale implications are not well understood (Wullschleger *et al*. 2002; Norby *et al*. 2004; Centritto 2005). This could offset some of the expected warming-induced increase in evaporative demand, thus easing the pressure for more irrigation water. This may also alter the relationship between meteorological drought and agricultural/hydrological drought; an increase in meteorological drought may result in a smaller increase in agricultural or hydrological drought owing to increased water-use efficiency of plants (Betts *et al*. 2007).

**Figure 11.**
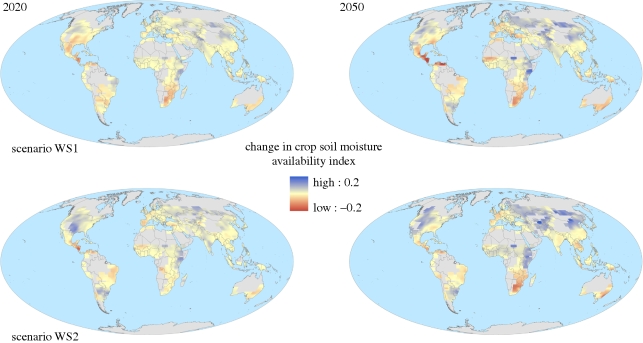
Two projections of future change in soil moisture as a fraction of that required to prevent plant water stress over global croplands for 30-year means centred around 2020 and 2050, relative to 1970–2000. Positive values indicate increased water availability. The two projections are the members of the ensemble with the greatest and least change averaged over all global croplands. See the electronic supplementary material for further details.

**Figure 12.**
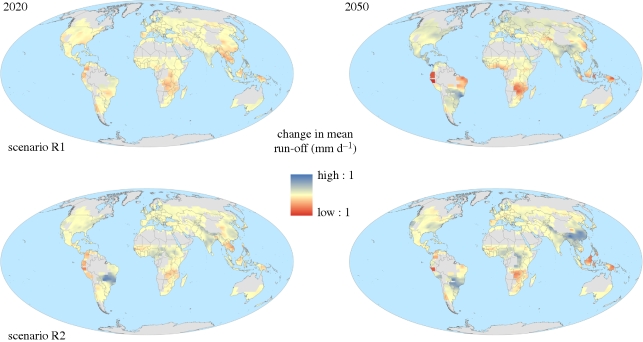
Two projections of future change in annual mean run-off (mm d^−1^) over global croplands for 30-year means centred around 2020 and 2050, relative to 1970–2000. The two projections are the members of the ensemble with the most positive and negative changes in annual mean run-off averaged over all global croplands. See the electronic supplementary material for further details.

Soil moisture and run-off may be more relevant than precipitation and meteorological drought indices as metrics of water resource availability, as these represent the water actually available for agricultural use. These quantities are routinely simulated by physically based climate models as a necessary component of the hydrological cycle. Figure 11 and table 1 show two scenarios of projected changes in soil moisture as a fraction of that required to prevent plant stress. The available soil moisture fraction is projected to increase on average across global croplands (table 1), with increases in some regions, particularly the mid-latitudes, but decrease in others, particularly in the tropics. Similarly, run-off increases in some regions and decreases in others (figure 12), but the mean change across global croplands varies in sign between scenarios (table 1). Importantly, the scenarios with an increase in mean run-off and the greatest increase in available soil moisture included the effects of CO_2_ fertilization in the model, while those with a decrease in mean run-off and the smallest increase in soil moisture availability did not include this effect (Betts *et al*. 2007).

**Figure 13.**
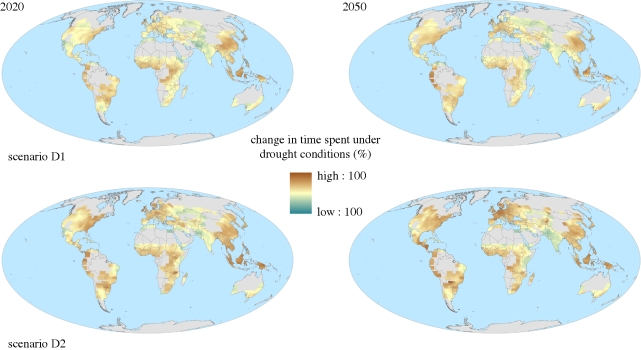
Two projections of percentage change in time spent under meteorological drought as defined in terms of soil moisture in global croplands for 30-year means centred around 2020 and 2050, relative to 2000. The two projections are the members of the ensemble with the greatest and least percentage change averaged over all global croplands. See the electronic supplementary material for further details.

However, as discussed in §2*b*, changes in extremes are also important, and agricultural drought may be more critical than annual mean soil moisture availability. With drought defined as the driest 20th percentile of the distribution in soil moisture over time in any given location, the model ensemble used here consistently projects an increase in the time spent under drought in most regions for the first half of the twenty-first century (figure 13 and table 1).

### (b) Ozone

Ozone is a major secondary air-pollutant, which at current concentrations has been shown to have significant negative impacts on crop yields (Van Dingenen *et al*. 2009). Whereas in North America and Europe, emissions of ozone precursors are decreasing, in other regions of the world, especially Asia, they are increasing rapidly (Van Dingenen *et al*. 2009).

Ozone reduces agricultural yield through several mechanisms. Firstly, acute and visible injury to products such as horticultural crops reduces market value. Secondly, ozone reduces photosynthetic rates and accelerates leaf senescence which in turn impacts on final yield. In Europe and North America many studies have investigated such yield reductions (e.g. Morgan *et al*. 2003). However, in other regions, such as Asia, little evidence currently exists. Thus, our understanding of the impacts in such regions is limited.

## 5. Conclusions

Anthropogenic greenhouse gas emissions and climate change have a number of implications for agricultural productivity, but the aggregate impact of these is not yet known and indeed many such impacts and their interactions have not yet been reliably quantified, especially at the global scale. An increase in mean temperature can be confidently expected, but the impacts on productivity may depend more on the magnitude and timing of extreme temperatures. Mean sea-level rise can also be confidently expected, which could eventually result in the loss of agricultural land through permanent inundation, but the impacts of temporary flooding through storm surges may be large although less predictable.

Freshwater availability is critical, but predictability of precipitation is highly uncertain and there is an added problem of lack of clarity on the relevant metric for drought—some studies including IPCC consider metrics based on local precipitation and temperature such as the Palmer Drought Severity Index, but this does not include all relevant factors. Agricultural impacts in some regions may arise from climate changes in other regions, owing to the dependency on rivers fed by precipitation, snowmelt and glaciers some distance away. Drought may also be offset to some extent by an increased efficiency of water use by plants under higher CO_2_ concentrations, although the impact of this again is uncertain especially at large scales. The climate models used here project an increase in annual mean soil moisture availability and run-off in many regions, but nevertheless across most agricultural areas there is a projected increase in the time spent under drought as defined in terms of soil moisture.

Moreover, even the sign of crop yield projections is uncertain as this depends critically on the strength of CO_2_ fertilization and also O_3_ damage. Few studies have assessed the response of crop yields to CO_2_ fertilization and O_3_ pollution under actual growing conditions, and consequently model projections are poorly constrained. Indirect effects of climate change through pests and diseases have been studied locally but a global assessment is not yet available. Overall, it does not appear to be possible at the present time to provide a robust assessment of the impacts of anthropogenic climate change on global-scale agricultural productivity.
